# Attitude toward contraception and abortion among Curaçao women. *Ineffective contraception due to limited sexual education?*

**DOI:** 10.1186/1471-2296-12-55

**Published:** 2011-06-23

**Authors:** Maaike J van den Brink, Adriana A Boersma, Betty Meyboom-de Jong, Jeanne GM de Bruijn

**Affiliations:** 1 Department of General Practice, University of Groningen, the Netherlands; 2 General Practice, Breedestraat (O) 33-35, Willemstad, Curaçao; 3 Department of General Practice, University of Groningen, the Netherlands; 4 Department of Sociology, University of the Netherland Antilles, Willemstad, Curaçao

## Abstract

**Background:**

In Curaçao is a high incidence of unintended pregnancies and induced abortions. Most of the induced abortions in Curaçao are on request of the woman and performed by general practitioners. In Curaçao, induced abortion is strictly prohibited, but since 1999 there has been a policy of connivance. We present data on the relevance of economic and socio-cultural factors for the high abortion-rates and the ineffective use of contraception.

**Methods:**

Structured interviews to investigate knowledge and attitudes toward sexuality, contraception and abortion and reasons for ineffective use of contraceptives among women, visiting general practitioners.

**Results:**

Of 158 women, 146 (92%) participated and 82% reported that their education on sexuality and about contraception was of good quality. However 'knowledge of reliable contraceptive methods' appeared to be - in almost 50% of the cases - false information, misjudgements or erroneous views on the chance of getting pregnant using coitus interruptus and about the reliability and health effects of oral contraceptive pills. Almost half of the interviewed women had incorrect or no knowledge about reliability of condom use and IUD. 42% of the respondents risked by their behavior an unplanned pregnancy. Most respondents considered abortion as an emergency procedure, not as contraception. Almost two third experienced emotional, physical or social problems after the abortion.

**Conclusions:**

Respondents had a negative attitude toward reliable contraceptives due to socio-cultural determined ideas about health consequences and limited sexual education. Main economic factors were costs of contraceptive methods, because most health insurances in Curaçao do not cover contraceptives. To improve the effective use of reliable contraceptives, more adequate information should be given, targeting the wrong beliefs and false information. The government should encourage health insurance companies to reimburse contraceptives. Furthermore, improvement of counseling during the abortion procedure is important.

## Background

There is a high incidence of unintended pregnancies and induced abortions in Curaçao. Most of the induced abortions are performed by general practitioners on request of the woman and not on medical indication [1].

In Curaçao, induced abortion is strictly prohibited, but since 1999 there has been a policy of connivance. Registration shows an abortion rate of 38, which is comparable to women from the Dutch Antilles living in the Netherlands (41.6)[2] (*Boersma AA, Alberts JF, Meyboom-de Jong B, Kleiverda G. 2010; unpublished data)*. The abortion rate is the number of abortions per 1000 women aged 15-45 years. In the Netherlands, where abortion is legalized since the eighties, live as much Dutch Antilleans as in Curaçao.

The rate is at least 8 times higher than among native Dutch women (5.3). Previous research on induced abortion among Dutch Antillean women in the Netherlands showed that induced abortion is associated with no use or ineffective use of contraception [3-6]. A Curaçao study on relationships, sexual behavior and Aids indicated the same phenomenon of no use or ineffective use of contraception, notwithstanding sexual activity [7].

Previous research has shown that socio-cultural as well as economic factors are related to inadequate sexual education and ineffective use of contraception. Socio-cultural factors like a taboo on discussing sexuality leads to poor sex education, and therefore to inadequate or limited knowledge of sexuality and contraceptives [7-10]. In cultures where males do not feel responsible for family planning, discussing contraception can be a problem especially when the male partner has a negative attitude toward the use of male contraceptives, e.g. condoms [6-11]. Another socio-cultural factor could indirectly be the predominant matrifocal household, where males are only temporarily present and relationship building between young couples is rare [12-14]. Previous research has shown associations between economic factors and no use or ineffective use of contraceptives. Low SES is known to be of important influence: lower education level in combination with uncertain future perspectives often results in minimal motivation to overcome the (alleged) disadvantages of reliable contraceptive methods [5-7,11]. Costs of contraceptives - often in combination with socio-cultural inhibitions - are also associated with no use or inadequate use [15]. The aim of this study has been to obtain information directly from women in Curaçao and to describe the risk factors of no or inconsistent contraceptive use by studying knowledge of and attitudes toward contraception and abortion, as well as the incidence of no use or inconsistent use of contraceptives related to its socio-cultural and economic antecedents.

Associations addressed in this article are between no or inconsistent use of contraceptives (dependent variable), and socio-cultural and economic antecedents based on literature, such as taboos on discussing sex, poor and wrong information on contraceptives, cost and the medical aspects of contraception.

## Methods

A research was performed among women during the period July to September 2009 visiting three general practices in Willemstad, the capital of Curaçao. The purpose was to investigate the knowledge and attitudes toward sexuality, contraception and abortion and the reasons for ineffective use of reliable contraceptives among women.

In each of the three participating general practices, the receptionist asked women between 15-45 years of age in the waiting room if they wanted to cooperate in a study by means of an interview performed by a sixth grade medical student. If the response was positive, the receptionist explained the interview was about contraception and abortion and would take place in a separate room. If their response was negative she asked for the reason of the refusal. For every interview, a structured questionnaire was used and completed by the same interviewer in a separate room. This method was chosen following results of recent research in Curaçao that in case of choice, lower educated and older respondents prefer face to face interviews above self report questionnaires. (*N.van Wijk, Data administration modes and social desirability bias, 2010 unpublished data*)[16].

The questionnaire was developed from two existing, validated questionnaires: "Sexual Health 2009", developed by the Nisso Rutgers Group and used in the Netherlands among others for Antillean women living in the Netherlands and "Codebook Marriage and Health", developed in Curaçao and used in publications in the Netherlands Antilles as well as in the Netherlands [7,11]. The two questionnaires were not used in their entirety. Some parts were irrelevant for the local situation and for the research questions. There were also some questions in common, where we had to choose between one of the two questionnaires. Furthermore, we added some questions about the cultural context of the interviewed women and some on abortion. The questionnaire is added as a separate file (Additional file 1).

The questionnaire was evaluated by two health professionals, experts in the field of sexuality and with experience in epidemiology, on completeness according to our research questions and translated from Dutch into the local language, Papiamento, by two translators, both working in the medical field. The sensitiveness of the subject made it important to translate the questions in a proper and a respectful manner. Discussing this with the mentioned professionals the most appropriate translation for each item has been chosen. A Dutch teacher working at the Fundashon di Planifikashon di Papiamento and a teacher Papiamento translated it, independently from each other, back from Papiamento in Dutch.

After four pilot interviews the professionals, the translator and the interviewer evaluated the questions were clear and understandable. Consensus was reached about the final formulation. Approval from the Medical Ethical Committee of Curaçao has been obtained.

During the interview, women were encouraged to explain their answers to the questions and to express their opinions and thoughts. In this way they were able to motivate their answers. In case somebody was unable to answer a question, no further suggestions were given and the answer was quoted as 'do not know'. Permission was asked to record the interview and participants were informed that participation was anonymous and confidential. Of the participating women, background characteristics like age, marital status, education, health insurance and employment were registered. The questionnaire about sexuality, contraception and abortion consisted of 49 questions (Table 1).

**Table 1:** Sections and themes of the interview questions about sexuality, contraception and abortion

Section	Multiple Choice questions	Open questions
**Sexuality (knowledge)**		
Sexual education	2	0
Knowledge of fertility	1	0
**Sexuality (attitude)**		
Sexual contacts	2	1
**Contraception (knowledge)**		
Oral contraception	5	0
Other contraceptive methods	3	0
**Contraception (attitude)**		
Importance of contraception	2	2
Disadvantages	2	4
Unplanned pregnancy	1	2
**Contraceptive use**	2	2
**Abortion**		
Personal	6	2
Knowledge	5	0
Attitude	4	1

### Sexuality and contraception: knowledge and attitude

The knowledge of sexuality was evaluated by 3 questions about source and quality of sexual education, e.g. "when are your fertile days?" (1 to 5 - all days not menstruating, just after menstruation, in between two menstruations, just before the menstruation or I don't know) and the attitude toward sexuality also by 3 questions e.g. "at what age did you have sex for the first time?"

The knowledge of contraception was tested by eight statements, e.g. "OCP use can make a woman infertile" (1 = yes, 2 = no, 3 = do not know).

Attitude toward contraception was assessed by thirteen items addressing:

a. importance of contraception, e.g. "what did you do when you realized you did not use your contraceptive in a proper way?" ( 1 = nothing, 2 = went to house physician, 3 = ECP, 4 = something else).

b. disadvantages of contraception, e.g. "OCP is not reliable enough" (1 = yes, 2 = no, 3 = do not know).

c. unplanned pregnancies and consequences, e.g. "in the previous six months did you have sex without using a contraceptive method?" (1 to 5 - never to very often).

### Contraceptive use

Contraceptive use was assessed by 4 questions addressing the way of protecting for unplanned pregnancy, e.g. "did you use a contraceptive method?" (1 = yes, 2 = no)

### Abortion: personal, knowledge and attitude

There were 7 personal questions about abortion, e.g. "Did you ever had an abortion?" Knowledge of abortion was acquired by 5 statements, e.g. "Abortion is a risky procedure" ( 1 = yes, 2 = no, 3 = do not know). Attitude toward abortion was assessed by 5 questions, e.g. "If abortion is legalized, the number of abortions will increase rapidly (1 = yes, 2 = no, 3 = do not know).

### Analysis of the results

Of the 49 questions about sexuality, contraception and abortion 35 were multiple choice. The respondent characteristics were divided in categories and the percentage of women was calculated for each category. We compared our respondent characteristics with the data of the Central Bureau of Statistics Netherlands Antilles [17].

For analysis of the results Microsoft Office Excel 2007 and the statistical program Statistical Package for the Social Sciences (SPSS), version 15.0 was used.

## Results

The response rate was 92%. Of the 158 women who were invited to participate, 146 women completed the questionnaire; 12 women did not participate giving lack of time as the main reason. Characteristics of respondents are presented in table 2. The responses of the 14 open questions were categorized by the first author. The alternatives offered by the women on the open questions are shown in table 3. Descriptive statistics of statements concerning contraceptive methods, contraceptive use and abortion are presented in table 4, 5 and 6.

**Table 2:** Characteristics of research population (n = 146)

Characteristics		Percentage [Number]
**Education**	Low^1^	10% [14]
	Medium^2^	76% [111]
	High^3^	11% [16]
	Unknown	3% [5]
**Employment**	Unemployed or voluntary service	9% [13]
	Pupils/Students	35% [52]
	Industrial workers	11% [16]
	Tradeswomen	3% [4]
	Office clerks and shop employers	30% [44]
	Professionals/directors/managers	12% [17]
**Health**	No insurance	3% [4]
**Insurance**	Pro-Pauper Insurance ( PP)^4^	17% [25]
	Social Insurance Security Bank (SVB)	67% [98]
	Civil Servant Health Insurance	5% [8]
	Other/Private Health Insurance	8% [11]
**Marital Status**	Single	29% [42]
	Partner	44% [64]
	Living together with partner	11% [16]
	Married	16% [24]
**Place of birth**	Curacao	79% [116]
	Dominican Republic	7% [10]
	The Netherlands	5% [7]
	Other island of the Netherlands Antilles	2% [3]
	Other	7% [10]

^1 ^Low: no education or primary education;

^2 ^Medium: pre-vocational and senior general secondary education, pre-university education;

*^3 ^High: university and higher vocational education*.

^4 ^Healthcare insurance paid by the government

**Table 3:** Open questions about sexuality, contraception and abortion

Sexuality	
1. With how many persons did you have sex in the last six months?	91.7%: one person; 5.8% two persons; 2.5% three persons
**Contraception**	
2. In case you didn't always use contraception: why didn't you do anything after having unprotected sex?	- 'I didn't know what to do', (low knowledge about emergency contraception), - 'I did not find it alarming to get pregnant' (will wait en see what to do once I get pregnant) - 'I thought the chance to become pregnant was very small' (for example by counting)
3. Why didn't you use always contraception in the last six months?	- Feeling side effects (esp. OCP), problems with taking pills every day - Resistance against hormones in pills and injectables, unnatural cycle - Easier to use nothing, fear of remaining rests of pills in the uterus or abdomen causing infertility or cancer - Costs and unplanned pregnancy not seeing as alarming
4. If you have experience with OCP, what disadvantages did you have?	- Side effects: Gain weight, nausea, vomiting, abdominal pain, stomach pain, depressed feelings, spotting, headaches, dizziness, shortness of breath - Other: forgetting pills, taking pills every day, not reliable, dangerous for health
5. If you have experience with condom use, what disadvantages did you have?	Possibility of rupture, less pleasure in sex
6. If you have experience with IUD, what disadvantages did you have?	Increase in menstrual fluid, pain during menstruation
7. If you have experience with injectable contraceptives, what disadvantages did you have?	Gain weight, nausea, vomiting, abdominal pain, stomach pain, spotting, headaches
8. Have you been unplanned pregnant? How often?	- 42% of participants: 0 unplanned pregnancies; - 35%: 1 unplanned pregnancy; - 14%: 2 unplanned pregnancies; - 5%: 3 unplanned pregnancies; - 4%: 4-7 unplanned pregnancies
9. What did you do when you were unplanned pregnant?	Abortion: 60%; nothing: 40%
**Contraceptive use**	
10. How did you protect yourself against pregnancy in the last six months?	Nothing: 24.6%; OCP: 29.5%; Condom 11.3%; Injectable contraceptive: 6.3%; Sterilization: 5.6%; IUD: 4.9%; PA and/or CI: 18.2%
11. In the past did you use (other) contraceptive methods? If yes, which?	OCP: 73%; Condom: 71%; CI: 45%; PA: 25%; Injectable contraceptive: 25%; IUD: 19%; EMC: 9%
**Abortion**	
12. When do you think abortion should be allowed?	- Never: 26.5%; - On request of the woman: 26.5%; - In exceptional cases: 47%, most mentioned were: after rape, fetal disabilities, health problems of woman, financial reasons, unwanted child.
13. At what age did you have your first abortion?	15 - 41 years; (mean age: 23 years)
14. Did you have contact with a healthcare provider after your abortion and what kind of healthcare provider?	72%: doctor; 19%: nobody, 9%: gynaecologist, psychologist, nurse

**Table 4:** Statements concerning knowledge of Contraceptive Methods (n = 146)

Statement	Agree (%)	Disagree (%)	? (%)
1. A man doesn't reach a orgasm during sexual intercourse. Still, the woman can become pregnant.	50	34	16
2. It is unhealthy to use the oral contraceptive pill for a period longer than 10 years.	44	14	42
3. If a woman uses the oral contraceptive pill, she can become infertile.	22	60	18
4. If a woman uses the oral contraceptive pill, her desire for sex becomes less.	7	73	20
5. The oral contraceptive pill is the most reliable contraceptive method.	49	43	8
6. The injectable contraceptive contains hormones, just as the oral contraceptive pill.	63	6	31
7. An intrauterine device can reside in the uterus without problems for 5 years.	42	20	38
8. A condom is not reliable because it ruptures rapidly.	39	53	8

**Table 5:** Contraceptive use in the past six months (n = 142*)

Contraceptive method	Total (n) [%]	Safe use (n)	Unsafe use (n)
No use of contraception	35 [24.6%]	22^1^	13
Oral Contraceptive pill (OCP)	41 [29.1%]	31	10
Condom	16 [11.3%]	6	10
Injectable contraception	9 [6.3%]	9	0
Sterilization	8 [5.6%]	8	0
Intra Uterine Device (IUD)	7 [4.9%]	7	0
Traditional methods^2^	26 [18.2%]	0	26
Total	142 [100%]	83 [58%]	59 [42%]

* *4 women who never had sexual intercourse are excluded from the analysis*.

^*1 *^*pregnancy wish, infertility, no sexual intercourse*

^2 ^Including Coitus Interruptus and/or Periodic Abstinence (counting) sometimes alternating with condom use

**Table 6:** Statements concerning abortion (n = 145)

Statement	Agree (%)	Disagree (%)	? (%)
1. Abortion is a good method of contraception.	2	89	9
2. In countries were abortion is legal are less complications after the abortion procedure.	27	32	41
3. Abortion is a risky procedure.	91	8	1
4. Abortion is easier than using the oral contraceptive pill, condoms or intrauterine device.	2	94	4
5. Abortion is hazardous for your body.	86	6	8
6. Abortion can cause infertility.	47	20	33
7. If abortion is legalized, the number of abortions will increase rapidly.	67	17	16

### Sexuality and contraception: knowledge and attitudes

According to 82% (120/146) of the respondents, they had received sufficient to comprehensive information about sexuality and contraception in the past. Only 18% considered the information they had received as insufficient, especially the information concerning contraceptive methods.

87% mentioned school as the most important source of information, followed by parents (51%), particularly the mother. In the third place, other sources such as the internet, books, seminars, television and information from other family members were mentioned (46%). Doctors, friends and partners were mentioned less frequently as sources of information (resp. 36, 27 and 26%).

The knowledge of respondents concerning contraceptive methods is presented in table 4. The main results show that half of the respondents knew that a woman can become pregnant using coitus interruption (CI) as a contraceptive method (50%). Almost half thought using the oral contraceptive pill (OCP) for a long period was harmful (44%), one fifth thought the OCP caused infertility (22%), and only half of respondents believed that the OCP is the most reliable contraceptive method (49%).

### Contraceptive use: incidences and antecedences

If we count all the respondents that mentioned risk behaviors, such as only using CI and/or Periodic Abstinence (PA) as a contraceptive or not using reliable contraceptives or inconsistent use of condoms or OCP, then 42% (59/142) of the respondents ran the risk of an unplanned pregnancy (Table 5).

10 of the 16 condom-users and 10 of the 41 OCP-users had not always used their contraceptive method during sexual intercourse in the past 6 months. A quarter (n = 35) used no method of contraception during the last 6 months. 13 because of (alleged) side effects, negative health considerations, costs (OCP cost per stripe in Curacao $ 5,- to $ 12,- a month, health insurances do not pay for contraceptives) or user problems, the remainder because of pregnancy wish, no sexual intercourse or infertility. In addition, there was a considerable number of women who used a traditional, less reliable method of contraception: 18% (26/142) applied (a combination of) counting, CI or an alternative method, sometimes alternating with condom use. These traditional methods were most used in first sexual relationships.

73% of respondents had used the OCP once in their life. As antecedences for no further use, two-thirds of the women mentioned having experienced side effects, mainly headache, stomach pain, weight increase, nausea and vomiting, and they considered OCPs expensive. Some of them had problems with taking the OCP every day, especially in the case of irregular sexual contact.

71% of the women had experience with condom use. The disadvantages mentioned were the possibility of rupture, and the experience of less pleasure during sexual contact.

25% of the respondents had used injectable contraceptives. The experienced side effects were comparable to use of the OCP, but injection was considered an easier method.

19% of respondents had ever used an IUD (Intra-Uterine Device). The problems experienced were an increase in menstrual fluid and pain attributed to the copper device. The hormonal device was generally more satisfactory for women.

9% of respondents used emergency contraception once in their life.

### Abortion: knowledge and attitudes

40% (58/146) of the respondents had a history of abortion: 28% one abortion and 12% more than one (range: 2-4). Age at the time of abortion varied from 15 to 41 years. Together, these 58 respondents underwent 82 abortions, including 47 medically-induced abortions, 32 surgical abortions and 3 self-induced abortions using herbs, Guinness Stout or a misprostol pill.

Considering the high incidence of abortion among the respondents, the attitudes toward abortion gave a very contrasting result: almost all respondents thought that abortion is a risky procedure (91%) and harmful for the body (86%). In contrast to what they practice, most of respondents did not see abortion as a method of contraception (89%) (Table 6).

Two thirds of the 58 respondents with experience of abortion were dissatisfied with having the abortion(65%). Almost two third experienced problems after the abortion procedure (57%), mainly emotional, but also physical problems or problems in their relationship or with their family. 72% had a check-up appointment after the abortion procedure and 9% had additional contact with healthcare providers concerning help for physical or emotional problems. Looking back, more than a quarter of the respondents wished they had received additional healthcare and/or help for emotional problems as well as more information about the abortion procedure and about contraceptive methods (28%).

## Discussion

Risk factors for no or inconsistent contraceptive use were mainly socio-culturally determined. Our findings show that the contraceptive knowledge of half of the respondents was either limited or just wrong, and their attitude toward reliable modern contraceptive methods was negative: half of the respondents mentioned fear of side effects and fertility problems and expressed their beliefs about the unreliability of OCP, safety doubts about the IUD, and rumors relating to the unreliability of condom use.

Quite remarkable was the frequent switch and temporary stops in the use of contraceptive methods, due to (alleged) side effects or to other supposed disadvantages mentioned by respondents. In our research group, there was a high frequency of side effects experienced with the OCP (73%). This is in strong contrast with pharmacotherapeutic literature that reports few side effects, and usually only in the first months after starting the OCP [18]. The percentage of respondents reporting the use of a contraceptive method was comparable with The Netherlands, but the effective use was much lower. Almost half of the respondents risked an unplanned pregnancy because of inconsistent contraception use or by using CI or PA as the contraceptive method.

Self-reported antecedents for inconsistent or no use were mostly socio-cultural in origin: user problems (taking pills), (alleged) side effects and negative health beliefs such as an unnatural menstrual cycle, resistance against hormone use, fear of infertility or the unreliability of contraceptives. Other socio-cultural antecedents given by our Curaçao women as reasons for not using modern contraceptives were no stable relationship, and therefore sexual contacts on an irregular basis. In the case of inconsistent condom use, the negative attitude of the male partner to condom use was mentioned as an important factor. The findings that ineffective use of contraception is related to poor sex education, little or incorrect knowledge of sexuality and contraception has been well-documented [19,20]. School education in Curaçao is on a good level and illiteracy is relatively low, but socio-cultural influences make it difficult to insert proper sexual education programs in the curriculum of (primary) schools [17].

Even if the effective use of contraception increases, the need for abortion will remain [21,22]. Therefore, the abortion policy and law should be modernized, and taken out of the criminal sphere. Safe abortion procedures and adequate information are important to lower the risk of complications for women.

Our research group is reasonably representative of the female population in Curaçao between age 15-45. There is a slightly lower percentage women with higher education in the research group (RG = 11%, pop. 12%), women with an employers insurance show quite an over-representation (RG = 67%, pop. 44%), and women with no insurance show some under-representation (RG = 3%, pop. 8%)[17]. There were some limitations in our study. The current questionnaire was not validated and not tested on reliability. The face to face method of interviews could have led to social desirable answers. On the other side non-response is very low in face to face interviews, which is positive in research on sensitive subjects like abortion and contraceptives. And only women attending three general practices in the centre of the capital were enrolled in the study. Including women visiting practices in the suburbs or the rural area might have given other results, although people in Curacao tend to seek the most appropriate general practitioner for them so most practices consist of a mixed population.

## Conclusion

In the case of no use or inconsistent use of contraceptives, socio-cultural factors of the population under review play an important role. Although the results of this study cannot be extrapolated to other Caribbean countries, it provides an insight into the problem of unplanned pregnancies caused by a lack of sexual education. In policy terms, we conclude that there is a strong need for specific sex education and contraceptive knowledge in order to improve the effective use of contraceptives and reduce the high rate of induced abortions. A special policy focus should be on programs in schools to talk about youth and (sexual) relationship building in order to promote safer sex, directed toward beliefs and attitudes. Besides sexual education in schools on a group level, adequate counseling concerning reliable, modern contraceptive methods to individual patients by healthcare providers is important. Economic elements are of less importance, although in all societies with gender inequality, governments should consider reimbursement for contraceptives as a method of reducing abortion rates.

Although the incidence of abortion in Curaçao is high, most respondents saw abortion as a risky procedure and not as a good alternative to contraception. Better social and medical counseling during the abortion procedure is a need identified and expressed by many women in the research group. Feelings of regret and guilt dominated, and additional help for emotional and physical problems and more information about the abortion procedure and effective contraceptive methods are essential.

## Abbreviations

SES: Social Economic Status; CI: Coitus Interruptus; OCP: Oral Contraceptive Pill; PA: Periodic Abstinence; IUD: Intra Uterine Device.

## Competing interests

The authors declare that they have no competing interests.

## Authors' contributions

MJvB collected and analyzed the data and wrote the manuscript, and AAB contributed to the analysis of the data and drafting of the manuscript. BMdJ and JGMdB were involved in the interpretation of the data and the critical review of the manuscript. All authors read and approved the final version.

## Pre-publication history

The pre-publication history for this paper can be accessed here:

http://www.biomedcentral.com/1471-2296/12/55/prepub

## Supplementary Material

Additional file 1 **Questionnaire Contraception and Abortion**. questionnaire with 64 questions: 8 demographic questions, 7 questions about pregnancy and children, 12 questions about knowledge of sexuality and contraception, 19 questions about sexuality and the use of contraception and 18 questions about knowledge of and attitude toward abortion. Click here for file
