# Supplementary material for: Attitude toward contraception and abortion among Curaçao women. Ineffective contraception due to limited sexual education?
Source: BMC Fam Pract. 2011 Jun 23;12:55. doi: 10.1186/1471-2296-12-55 (PMC3146412; doi:10.1186/1471-2296-12-55)
Supplement: Additional file 1 — Questionnaire Contraception and Abortion. questionnaire with 64 questions: 8 demographic questions, 7 questions about pregnancy and children, 12 questions about knowledge of sexuality and contraception, 19 questions about sexuality and the use of contraception and 18 questions about knowledge of and attitude toward abortion. [file 1471-2296-12-55-S1.DOC]

**Questionnaire Contraception and Abortion**

This questionnaire includes the following topics: sexuality, pregnancy, contraception and abortion. We find it important to know what your knowledge of and attitude toward contraception and abortion is. We also would like to know which method of contraception you use.

**General**

**1. What is your age?** … years old.

**2. What is de highest level of education you have finished?**

- None
- Primary school
- Lower vocational education
- Secondary education
- Secondary vocational education
- High school
- Higher vocational education
- Higher general education
- University

**3. Do you have a paid job? If so, what kind of job do you have?**

- No
- Yes, being…

**4. Do you have a health Insurance? If so, which health insurance?**

- No
- Yes, being …
- Pro-Pauper Insurance (PP)
- Social Insurance Security Bank (SVB)
- Civil Servant Health Insurance

Private Health Insurance:

- Fatum
- Ennia
- Citizen Insurance
- Alico
- Other, being ...

**5. What kind of relationship do you have?**

- Single
- Single with incidental sexual contacts
- Partner, since … months / ... years
- Partner, since … months / ... years and also incidental sexual contacts
- Married or living together with partner
- Married (or living together with partner) and also other incidental sexual contacts

**6. Have you been born in Curaçao?**

- No
- Yes 🡪 *go to question 9*

**7. If not, were have you been born?**

- Other island of the Netherlands Antilles
- The Netherlands
- Dominican Republic
- Colombia
- Jamaica
- Other country in Europe
- Other country in the Caribbean
- Other country in South America
- Other country in the world

**8. For how long have you been living in Curaçao?** … years

**Pregnancy and Children**

The following questions concern pregnancy and children.

**9. Have you ever been pregnant? Think also about terminated pregnancies (abortion/miscarriage)**

- No *🡪 go to question 14*
- Yes, ... time (s) (in total)

**10. Do you have children?**

- No *🡪 go to question 14*
- Yes

**11. How many children do you have?** Number: …………

**12. From how many fathers?** Number: ……………

**13. How old were you when you had your first child?** … years old.

**14. How important is it for you to have children?**

- Unimportant
- Not that important
- Important
- Very important

**15. What is your opinion about the following statement?**

**‘A woman is only a real woman when she has children’.**

- Totally disagree
- Partially disagree
- No opinion
- Partially agree
- Totally agree

**Knowledge of sexuality and contraception**

The following questions are about knowledge of and attitude toward sexuality and contraception. Contraceptive methods are all methods you can use to prevent a pregnancy.

**16. Did you have sexual education? For example: information about safe sex and contraceptive methods.**

- No, no education 🡪 *go to question 18*
- Yes, but insufficient information
- Yes, sufficient information
- Yes, extensive information

**17. Who gave you this information? More than one answer is possible.**

- parents
- friends
- partner
- school
- governmental campaigns
- doctor or other health care providers?
- other, being ...

**Knowledge of you own body**

**18. When are your fertile days?**

- All days when I don’t menstruate
- Just after my menstruation
- In between my menstruations
- Just before my menstruation
- I do not know

**Knowledge of contraception**

**The following statements are about the use of contraceptive methods.**

**What do you think? Are the statements correct (C), incorrect (I) or ‘I don’t know’ (?)**

**19.** A man does not reach an orgasm during sexual intercourse.

Still, the woman can become pregnant. C I ?

**20.** It is unhealthy to use the oral contraceptive pill

for a period longer than 10 years. C I ?

**21.** If a woman uses the oral contraceptive pill, she can become infertile. C I ?

**22.** If a woman uses the oral contraceptive pill, her desire for sex is less C I ?

**23.** The oral contraceptive pill is the most reliable contraceptive method. C I ?

**24.** The injectable contraceptive contains hormones,

just as the oral contraceptive pill. C I ?

**25.** An intrauterine device can reside in the uterus without problems C I ?

for 5 years.

**26.** A condom is not reliable because it ruptures rapidly. C I ?

**27. How important do you find the use of a contraceptive method?**

- Totally unimportant
- Unimportant
- No opinion
- Important
- Very important

**Sexuality and Contraception**

The following questions are about sexuality and the use of contraceptive methods.

**28. At which age did you have sexual intercourse with a man for the first time?**

- Never *🡪 go to question 47*
- Age: ... years old

**29. Did you have sexual intercourse in the past six months?**

- No *🡪 go to question 31*
- Yes

**30. If so, with how many persons? (If you do not know the actual number, an estimation is sufficient)**

- With ... persons

**31. Did you use a method to prevent pregnancy in the past six months?**

- No 🡪 *go to question 33*
- Yes

**32. If so, in what way did you prevent pregnancy? (More answers are possible and please note: sterilization is also a method to prevent pregnancy)**

- Periodic abstinence (no sexual intercourse during your fertile period)
- Withdrawal ( coitus interruptus)
- Condom
- Oral contraceptive pill (or minipill)
- Condom and oral contraceptive pill
- Injectable contraceptive
- The contraceptive patch(Evra)
- Contraceptive implants (Implanon)
- Contraceptive ring (Nuvaring)
- Pessarium (rubber koepeltje)
- Copper intra uterine device
- Hormonal intrauterine device (Mirena)
- Sterilization partner
- Sterilization (woman)
- Morning After Pill
- Abortion by a doctor (for instance medication, curettage)
- Own methods to end the pregnancy (for example: medication from the pharmacy, herbs, Guinness Stout or other)
- Other, being ...

**33. Have you been using (other) contraceptive methods in the past?**

- No
- Yes, being …. (see options question 32)

**Answer the following questions if you have experience with the use of the oral contraceptive pill, condom, intrauterine device or the injectable contraceptive:**

**Which of the following disadvantages did you experience? More answers are possible.**

**34**. **Oral contraceptive pill**:

- The oral contraceptive pill is too expensive
- The oral contraceptive pill is not reliable enough (the risk to get pregnant is too high)
- The oral contraceptive pill has side effects (for instance headache, stomach pain, nausea)
- It is difficult to use the oral contraceptive pill in the right way
- Weight increase due to the oral contraceptive pill
- You can become infertile due to use of the oral contraceptive pill
- It is unhealthy to use the oral contraceptive pill
- Other disadvantages, being ...
- No disadvantages

**35.** **Condom**:

- Condoms are too expensive
- Condoms are not reliable enough (the risk to get pregnant is too high)
- It is difficult to use condoms in the right way
- It is difficult to buy condoms
- Using a condom gives less pleasure
- Putting on a condom is an unpleasant interruption during the sex
- You have to ask your (sexual) partner to use a condom
- Other disadvantages, being ...
- No disadvantages

**36.** **Intra uterine device (IUD):**

- My menstruation is more painful due to the IUD
- My menstrual fluid increases due to the IUD
- Bleedings during the menstruation free period caused by the IUD
- I have less or no menstruation due to the IUD
- I can feel the IUD
- My partner feels the IUD
- I have abdominal pain due to the IUD
- It is painful to have the IUD inserted
- I have to go to the doctor for insertion of an IUD
- Other disadvantages being ...
- No disadvantages

**37**. **Injectable contraceptive:**

- The injectable contraceptive has side effects
- Weight increase due to the injectable contraceptive
- Bleeding during the menstruation free period caused by the injectable contraceptive
- I have less or no menstruation due to the injectable contraceptive
- I have to go to the doctor for using the injectable contraceptive
- The injection is painful
- Other disadvantages, being ...
- No disadvantages

**The following questions are about the contraceptive method you are using currently.**

**If you are using no contraceptive method, go to question 41.**

**38. Are you satisfied with your method of contraception?**

- Totally unsatisfied
- Unsatisfied
- Neutral
- Satisfied
- Very satisfied

**39. Do you find it difficult to use your method of contraception in the right way?**

- Very difficult
- Difficult
- Neutral
- Easy
- Very easy

**40. Did you have sexual intercourse in the past six months without using a contraceptive method?**

- Never 🡪 *go to question 42*
- Once
- Multiple times
- Often
- Very often

**41. Why did you not (always) use a contraceptive method in the past six months? More answers are possible.**

- I have no sexual intercourse
- I have a pregnancy wish
- I do not mind becoming pregnant
- When I get pregnant, I will decide what to do
- I do not menstruate anymore (menopause)
- I am infertile or less fertile
- My partner is infertile / less fertile
- My religion does not allow it
- My partner does not want to use contraceptive methods
- It is difficult to use contraceptive methods
- I don’t know how to get contraceptive methods
- I’m afraid to buy contraceptive methods
- Contraceptive methods are too expensive
- Contraceptive methods are not effective
- Contraceptive methods have to many side effects
- If you use a contraceptive method, you can become infertile
- It is unhealthy to use contraceptive methods
- Other, being…

**42. Imagine: you did not use your contraceptive method in a proper way. What did you do when you discovered it? More answers are possible.**

- I didn’t do anything
- I went to the doctor or other health care provider *🡪 go to question 44*
- I used the morning after pill *🡪 go to question 44*
- I did something else, being … *🡪 go to question 44*

**43. Why did you not do anything? More answers are possible.**

- The risk I would be pregnant was small
- I thought it was too much effort ?
- I did not mind becoming pregnant
- My partner said he wanted a child
- My partner said he would support me if I would become pregnant
- I didn’t know what to do
- I felt guilty
- I felt ashamed
- Other, being …

**44. Have you ever had an unplanned pregnancy?**

- No *🡪 go to question 47*
- Yes, … time(s)

**45. Think about the last time you had an unplanned pregnancy. Was the pregnancy wanted?**

- No
- At first not, but later on it was wanted
- Yes, from the beginning of the pregnancy

**46. What did you do when you had an unplanned pregnancy?**

- I kept the child
- I had an abortion
- Other, being …

**Abortion**

De following questions are about abortion. Abortion is prematurely ending of a pregnancy due to medical intervention.

**47. Do you know people who had an abortion?**

- No
- Yes

**48. Did you ever had an abortion?**

- No  *🡪 go to question 55*
- Yes, … time(s)

**49. Which abortion method did you use?**

- curettage
- medication from a doctor
- self medication (without help of a doctor/ by myself)
- other, being ...

**50. How old were you when you had your first abortion?** … years old.

**51. Afterwards, are you satisfied with having the abortion? (If having more abortions, this question is about the last abortion)**

- No, I am not satisfied
- Neutral / More or less satisfied
- Yes, I am satisfied

**52. After an abortion, did you have any of the following complaints*?* More answers are possible.**

- Physical complaints
- Emotional complaints
- Sexual problems
- Family problems
- Relational problems
- Problems concerning employment or educational training
- Problems with pregnancy or giving birth
- Other, being ...
- I did not have problems or complaints

**53. Did you have contact with a doctor or other health care provider after your abortion?**

- No
- Yes, sort of health care provider:

**54. After your abortion, did you wish to have help or advice from someone like a doctor or other health care provider?**

- No
- Yes

**55. When do you think abortion should be allowed?**

- Never 🡪 go to question 57
- Only in exceptional cases
- Always (it is a individual choice of every woman) 🡪 go to question 57

**56. In which exceptional case do you think abortion should be allowed? You can choose multiple answers.**

- A congenital defect of the fetus
- Health risk for the mother
- Rape
- If the child is not wanted
- Social or financial reasons: an uncertain future for the child, no stability in the family for the child to grow up.
- Other, being …

**57. Abortion is prohibited by law in Curaçao.**

**What do you think about the following statement?**

**Abortion has to be legalized in Curaçao.**

- Totally disagree
- Partially disagree
- Neutral
- Partially agree
- Totally agree

**What do you think about the following statements concerning abortion? Do you agree (A), Disagree (D) or ‘you do not know’ (?) ?**

**58**. Abortion is a good method of contraception. A D ?

**59.** In countries were abortion is legal

are less complications after the abortion procedure. A D ?

**60.** Abortion is a risky procedure. A D ?

**61.** Abortion is easier than using the oral contraceptive pill,

condoms or intra uterine device. A D ?

**62.** Abortion is hazardous for your body. A D ?

**63.** Abortion can cause infertility. A D ?

**64.** If abortion is legalized, the number of abortions will increase rapidly. A D ?
